# Supplementary material for: Differences in Functional Capacity between Oncologic and Non-Oncologic Populations: Reference Values
Source: Healthcare (Basel). 2024 Jan 25;12(3):318. doi: 10.3390/healthcare12030318 (PMC10855221; doi:10.3390/healthcare12030318)
Supplement: Supplementary file 1 [file healthcare-12-00318-s001.zip › healthcare-2810152-supplementary.pdf]

Supplementary Table S1. Percentiles of MGS, kg of Muscle and 5STS rel.

		Percentiles						
**Women**	**Age group**	**5th**	**10th**	**25th**	**50th**	**75th**	**90th**	**95th**
MGS	G1=50-59 (n=73)	16.0	19.2	21.9	24.2	27.4	30.2	33.0
	G2=60-69 (n=58)	14.9	17.2	20.5	23.3	25.8	27.9	30.2
	G3=70-79 (n=18)	14.0	14.4	15.9	18.6	21.6	24.7	25.9
	G4=80-89 (n=3)	18.8	18.8	19.0	19.3	19.4	19.5	19.5
Kg_Muscle	G1=50-59 (n=73)	18.8	19.9	20.7	22.6	24.3	26.4	28.7
	G2=60-69 (n=58)	17.8	18.6	19.8	22.1	23.7	26.1	27.8
	G3=70-79 (n=18)	17.3	17.6	18.3	19.8	22.1	23.0	23.8
	G4=80-89 (n=3)	17.9	18.1	18.7	19.7	20.8	21.5	21.7
STS (s)	G1=50-59 (n=73)	19.3	16.0	14.1	12.7	11.0	9.7	8.8
	G2=60-69 (n=58)	23.7	18.9	15.2	13.1	11.6	10.5	10.1
	G3=70-79 (n=18)	24.0	23.4	18.8	14.3	12.6	11.5	11.0
	G4=80-89 (n=3)	32.2	30.7	26.2	18.7	16.2	14.7	14.2
5STS rel. (W·kg ^{1})	G1=50-59 (n=73)	1.4	1.6	1.9	2.2	2.5	3.0	3.2
	G2=60-69 (n=58)	1.2	1.4	1.7	2.0	2.3	2.6	2.7
	G3=70-79 (n=18)	1.0	1.086	1.40	1.71	2.0	2.2	2.6
	G4=80-89 (n=3)	0.8	0.9	1.0	1.3	1.6	1.8	1.9

Men

MGS	G1=50-59 (n=40)	29.9	32.4	39.6	44.3	47.8	53.0	57.8
	G2=60-69 (n=59)	29.3	31.1	35.7	39.10	45.7	49.9	51.6
	G3=70-79 (n=27)	23.7	25.8	29.4	33.7	36.9	40.6	41.4
	G4=80-89 (n=2)	25.8	26.1	27.2	29.1	30.9	31.9	32.2
Kg_Muscle	G1=50-59 (n=40)	25.6	26.4	29.4	32.4	37.1	39.7	40.9
	G2=60-69 (n=59)	24.8	25.7	28.1	31.6	34.4	35.9	37.0
	G3=70-79 (n=27)	20.1	22.5	24.9	27.2	30.6	31.9	33.3
	G4=80-89 (n=2)	26.6	26.7	26.9	27.3	27.8	27.9	28.0
STS (s)	G1=50-59 (n=40)	18.3	16.1	13.7	10.9	10.0	9.0	8.6
	G2=60-69 (n=59)	18.8	17.1	13.8	12.3	11.0	9.3	8.8
	G3=70-79 (n=27)	22.7	20.0	16.4	14.9	12.5	11.4	10.7
	G4=80-89 (n=2)	20.6	20.1	18.8	16.5	14.3	13.0	12.5
5STS_rel. (W·kg ^{1})	G1=50-59 (n=40)	1.7	2.1	2.5	3.1	3.5	3.9	4.0
	G2=60-69 (n=59)	1.9	1.9	2.3	2.7	3.1	3.6	3.7
	G3=70-79 (n=27)	1.3	1.6	1.9	2.2	2.4	2.7	2.8
	G4=80-89 (n=2)	1.4	1.5	1.6	1.9	2.2	2.4	2.4

MGS =Manual Grip strength, Kg_Muscle=Kilograms of muscle, 5STS =5 time Sit to Stand Test, 5STS_rel =Relative power
